# Supplementary material for: Haptics based multi-level collaborative steering control for automated driving
Source: Commun Eng. 2023 Jan 23;2:2. doi: 10.1038/s44172-022-00051-2 (PMC10955919; doi:10.1038/s44172-022-00051-2)
Supplement: Supplementary file 2 — Supplementary Information [file 44172_2022_51_MOESM2_ESM.pdf]

Haptics based Multi-Level Collaborative Steering Control for Automated Driving

Tomohiro Nakade, Robert Fuchs, Hannes Bleuler and Jürg Schiffmann

SUPPLEMENTARY NOTES 1:

There are two types of hands-on detection (HOD): capacitive and torque based [1]. Capacitive detection is conceptually ideal for detecting hands in contact with the steering wheel. It is particularly appropriate for hands-off detection. However, the hands-on state does not confirm that the driver is actively steering. Detection based on a steering torque sensor is appropriate for measuring steering activity from the driver. Therefore, it is relevant for hands-on detection. However, performance for detection of the hands-off state is limited because there is no unique relation with the driver torque. Furthermore, capacitive-type sensors mounted on the steering wheel rim do not satisfy the level of functional safety required by automotive standards.

SUPPLEMENTARY TABLE 1: DESCRIPTION OF SYMBOLS

Symbol	Description
$T_d,\ T_a$	Driver and automation torque
$Z_d,\ Z_a$	Driver and automation impedance
$\xi_d,\ \xi_a$	Driver and automation angle tracking error
$\theta_d,\ \theta_a$	Driver and automation target angle
$\theta_{sw},\ \theta_p$	Steering wheel and pinion shaft angle
T_{mot}	Motor torque command
ϵ	White noise in driver and automation torque input
Ψ	EPS dynamics
T_{tb}	Torsion bar torque output
T_{ld}	Nonlinear disturbance
θ_{cmd}	Inner loop angle command
θ_m	Manual deviation
κ	Interaction type setting parameter
$Z_{a,0}$	Nominal automation impedance
$\hat{Z}_d,\ \hat{\theta}_d$	Estimated driver impedance and target angle (goal)
$\theta_{env},\ \theta_{int}$	Driver environmental and intentional desired angle
v_x	Vehicle longitudinal velocity
ρ	Road curvature
$w,\ v$	Process and observation noise
K	Kalman filter gain
P	Solution of Riccati equation
$Q,\ R$	Co-variance matrix of process and observation noise
$F,\ H$	Jacobian matrix of system and observation equation
Δy_d	Driver desired lateral deviation
Δy_v	Lateral deviation from AD trajectory
β	Side slip angle
γ_m	Driver desired yaw rate
y_r	Candidate path polynomials
y_{rf}	Path candidate final position
t_f	Trajectory adaptation completion time
$y_{r,opt}$	Adapted lateral position reference
C_y	Cost function for trajectory adaptation
J_y	Jerk summation to complete trajectory adaptation
t_{sc}	Test duration of driver quantitative study

SUPPLEMENTARY TABLE 2: DESCRIPTION OF PARAMETERS

Parameter	Description	Value
i_s	EPS reduction gear ratio	18.5
J_{sw}	Steering wheel inertia	$0.0355\ kgm^2$
J_p	Lower part of torsion bar inertia	$0.0285\ kgm^2$
K_{tb}	Torsion bar stiffness	$162.72\ Nm\,rad^{-1}$
$T_{z,a}$	Automation impedance time constant	$0.01\ s$
$T_{z,d}$	Driver impedance time constant	$0.01\ s$
J_{vp}	Virtual EPS inertia	$0.0570\ kgm^2$
M_v	Vehicle mass	$2025\ kg$
I_z	Vehicle yaw moment of inertia	$2800\ kgm^2$
l_f	Distance from CG to front axles	$1.3\ m$
l_r	Distance from CG to rear axles	$1.6\ m$
C_f	Front cornering stiffness	$57000\ Nm\,rad^{-1}$
C_r	Rear cornering stiffness	$59000\ Nm\,rad^{-1}$
i_o	Steering overall gear ratio	16
J_d	Driver inertia	$0.0177\ kgm^2$
t_i	Propagation time for driver goal	$0.1\ s$
t_s	Trajectory adaptation sensitivity	$1\ s$
Δt	Sampling time for Kalman filter	$0.0004\ s$
k_j	Weight for jerk	0.03
k_t	Weight for completion time	17.3
k_a	Weight for automation error	1
k_m	Weight for driver error	50

REFERENCE

REFERENCES

[1] M. Moreillon, T. Tsutomu, and R. Fuchs, "Highly automated driving – detection of the driver's hand on and off the steering wheel for ADAS and autonomous driving," in *7th International Munich Chassis Symposium 2016*, P. D. P. E. Pfeffer, Ed. Wiesbaden: Springer Fachmedien Wiesbaden, 2017, pp. 505–525.
